# 638. Utility of Recombinant Hepatitis B Vaccine in Pre-liver Transplant Candidates

**DOI:** 10.1093/ofid/ofac492.690

**Published:** 2022-12-15

**Authors:** Jack Rogers, Pooja Prasad, Amy Weinberg, John S Bynon, Victor I Machicao, Masayuki Nigo

**Affiliations:** McGovern Medical School, Houston, Texas; McGovern Medical School at UTHealth Houston, Houston, Texas; McGovern Medical School at UT Health Houston, Houston, Texas; McGovern Medical School, Houston, Texas; McGovern Medical School, Houston, Texas; UT Health Mc Govern Medical School, Houston, Texas

## Abstract

**Background:**

A recombinant, adjuvanted Hepatitis B (HBV) vaccine (HepB-CpG) was approved by the FDA in 2017. Initial FDA data showed greater immunogenicity than comparable recombinant HBV vaccines. Moreover, it requires only two doses over four weeks instead of three doses over six months, so there is a time advantage to HepB-CpG, which is advantageous in pre-liver transplant candidates, given their limited time before transplantation. The efficacy in this population is unknown, so we explored its efficacy This study aims to explore the efficacy of HepB-CpG in pre-liver transplant candidates, as there is currently no data on this subject.

**Methods:**

This study was a retrospective review of pre-liver transplant candidates greater than 18 years old at Memorial Hermann Hospital in Houston, TX, who received at least two doses of HepB-CpG. Patients were identified from our pre-liver transplant database. Patient characteristics were collected at the time of the first dose. Vaccine efficacy was measured by anti-Hepatitis B surface antibody titers (≥10mIU/mL) at least four weeks after completion of the series. Patients who received only one dose or did not have any post-vaccine titers were excluded from the analysis.

**Results:**

A total of 78 potential eligible patients were identified. After exclusion due to various reasons, a total of 27 patients were eligible for our study. Data are summarized in Table 1. Briefly, our patients were Hispanic dominant (59.3%). The median MELD score was 18, and a significant number of patients (48.1%) had underlying malignancy. The cause of cirrhosis was mainly due to alcoholic and non-alcoholic steatohepatitis (NASH). Only 13 patients (48.1%) showed seroconversion after their second dose of HepB-CpG. Moreover, of the five patients receiving immunosuppressive drugs, only one patient (20%) responded to the vaccine.

Characteristics of Pre-Liver Transplant Candidates Who Had Serological Evaluation after Two Doses of Recombinant Adjuvanted Hepatitis B Vaccine.

**Figure:**
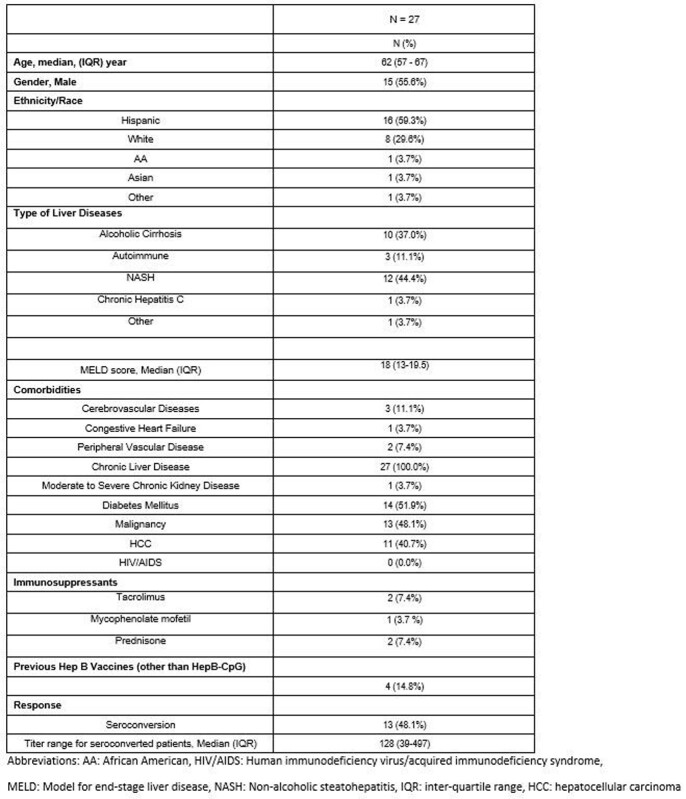

**Conclusion:**

Despite administration of HepB-CpG, less than 50% of pre-liver transplant candidates showed seroconversion. Furthermore, only one cirrhotic patient on immunosuppressive drugs responded to the vaccine. Our results illustrate the need for a better strategy to improve immunogenicity among this patient population. Larger studies are warranted to confirm the findings.

**Disclosures:**

**Victor I. Machicao, M.D.**, Gilead Inc: Advisor/Consultant.

